# Understanding patterns of family support and its role on viral load suppression among youth living with HIV aged 15 to 24 years in southwestern Uganda

**DOI:** 10.1002/hsr2.467

**Published:** 2022-01-19

**Authors:** Rachael Mukisa Nakandi, Patricia Kiconco, Angella Musiimenta, John Johnes Bwengye, Sylivia Nalugya, Richard Kyomugisa, Celestino Obua, Esther Cathyln Atukunda

**Affiliations:** ^1^ Mbarara University of Science and Technology Mbarara Uganda

**Keywords:** antiretroviral therapy adherence, disclosure, family relationships, social support, stigma, youth living with HIV

## Abstract

**Background:**

Active family support helps as a buffer against adverse life events associated with antiretroviral therapy (ART) uptake and adherence. There is limited data available to explain how family support shapes and affects individual healthcare choices, decisions, experiences, and health outcomes among youth living with HIV (YLWH). We aimed to describe family support patterns and its role in viral load suppression among YLWH at a rural hospital in southwestern Uganda.

**Methods:**

We performed a mixed‐method cross‐sectional study between March and September 2020, enrolling 88 eligible YLWH that received ART for at least 6 months. Our primary outcome of interest was viral load suppression, defined as a viral load detected of ≤500 copies/mL. Data analysis was performed using Statistical Package for Social Sciences version 20. Fifteen individuals were also purposively selected from the original sample and participated in an in‐depth interview that was digitally recorded. Generated transcripts were coded and categories generated manually using the inductive content analytic approach. All participants provided written consent or guardian/parent assent (those <18 years) to participate in the study.

**Results:**

Forty‐nine percent of YLWH were females, the median age was 21 (IQR: 16‐22) years. About half of the participants (53%) stayed with a family member. A third (34%) of participants had not disclosed their status to any person they stayed with at home. Only 23% reported getting moderate to high family social support (Median score 2.3; IQR: 1.6‐3.2). Seventy‐eight percent of YLWH recorded viral load suppression. Viral load suppression was associated with one living with a parent, sibling, or spouse (AOR: 6.45; 95% CI: 1.16‐16.13; *P* = .033), having a primary caretaker with a regular income (AOR: 1.57; 95% CI: 1.09‐4.17; *P* = .014), and living or communicating with family at least twice a week (AOR: 4.2; 95% CI: 1.65‐7.14; *P* = .003). Other significant factors included youth receiving moderate to high family support (AOR: 12.11; 95% CI: 2.06‐17.09; *P* = .006) and those that perceived family support in the last 2 years as helpful (AOR: 1.98; 95% CI: 1.34‐3.44; *P* = .001). HIV stigma (AOR: 0.10; 95% CI: 0.02‐0.23; *P* = .007) and depression (AOR: 0.31; 95% CI: 0.06‐0.52; *P* = .041) decreased viral load suppression. Qualitative data showed that dysfunctional family relationships, economic insecurity, physical separation, HIV‐ and disclosure‐related stigma, past and ongoing family experiences with HIV/ART affected active family support. These factors fueled feelings of abandonment, helplessness, discrimination, and economic or emotional strife among YLWH.

**Conclusion:**

Our data showed that living with a family member, having a primary caretaker with a regular income, living or communicating with family members regularly, and reporting good family support were associated with viral load suppression among YLWH in rural southwestern Uganda. Experiencing depression due to HIV and or disclosure‐related stigma was associated with increased viral load. All YLWH desire ongoing emotional, physical, and financial support from immediate family to thrive and take medications daily and timely. Future interventions should explore contextual community approaches that encourage acceptance, disclosure, and resource mobilization for YLWH who rely on family support to use ART appropriately.

## 1 INTRODUCTION

Young people (15‐24 years old) constitute the most significant population in the world and, at the same time, represent an age group with one of the highest new HIV infections.^1^ Young people are also at a greater risk of AIDS‐related deaths, discrimination, marginalization, exclusion, poor antiretroviral therapy (ART) adherence, and the lowest utilization of health care.^1^, ^2^ According to several studies, young people are faced with persisting barriers that negatively impact their access to services such as family or spousal consent requirements, family, economic and structural factors, social support, difficulties in transitioning from pediatric care to self‐management, criminalization against vital young populations, age, inadequate health systems, early and forced marriages, and a lack of appropriate sexual education.^1^, ^3^, ^4^, ^5^ In Uganda, individuals who are 10 to 24 years of age comprise 33% of the whole population and they account for the highest number of the country's HIV/AIDS cases.^6^ Most youth in Uganda are financially and emotionally dependent on their families, and studies have shown that the main barriers affecting their ART uptake and adherence include unreliable social support, change in guardianship, poverty, HIV‐ and disclosure‐related stigma, school attendance limiting their privacy, loss to follow‐up, drug side effects, and substance abuse, among others.^7^, ^8^, ^9^

Social support has been documented to improve medication adherence through emotional support (psychological and informational) and instrumental support (physical and economic), helping to overcome many physical, structural, and financial barriers to access care in time.^10^ Support from close family members could also make individuals feel a sense of security and belonging, facilitating them to overcome significant physical hurdles such as food insecurity, housework, child care, and transport challenges to access ART.^11^ Healthy family support is therefore vital for every individual's well‐being, acting as a source of stability, happiness, empathy, encouragement, connection, and a platform to express how one feels, celebrate good experiences, and talk about challenging times.^12^ This active family support also provides members with the much‐needed sense of identity that could ultimately help enhance the quality of life and adherence by providing a necessary buffer against adverse life events. However, negative family social support, socio‐cultural perception of HIV disease, stigma, poor relationships from unsupportive family members, non‐communication, mistrust, resentment, abandonment, or loose family ties can affect the pattern of health‐seeking, pill‐taking behavior, coping mechanism against HIV‐related stigma, and well‐being of an individual as a whole.^10^, ^13^, ^14^

Several studies have attempted to explore the effect of social support on medication adherence.^10^, ^15^ However, limited data explain how support from family members explicitly shapes and affects individual healthcare decisions, experiences, and health outcomes among youth living with HIV (YLWH). This study aims to describe patterns of family support and how it affects viral load suppression among youth aged 15 to 24 years in southwestern Uganda.

## 2 METHODS

### 2.1 Study design and setting

We conducted a cross‐sectional study to describe the patterns of family support and its effect on viral load suppression among youth aged 15 to 24 years in southwestern Uganda. The study was conducted at the Kinoni Health Center IV, a publically funded and operated health center in Rwampara, a rural, resource‐limited district located in southwestern Uganda. The health center serves over 100 000 patients annually from across 20 villages. It provides general outpatient care, maternal and child health care, inpatient care, general surgery, laboratory diagnostics, and HIV care services to both children and adults.

### 2.2 Study population

The study was conducted among YLWH between 15 and 24 years of age, both male and female, registered at the ART clinic of Kinoni Health Centre IV.

### 2.3 Sampling procedure and recruitment

We selected all study participants who had attended the HIV/ART clinic for the last 6 months as per the facility records assisted by the nurse‐in‐charge for the required age group. We enrolled youth between 15 and 24 years of age, both male and female, living with HIV and registered at Kinoni Health Centre IV for HIV/ART care. Eligible participants should have been enrolled on ART for at least 6 months. We excluded individuals who declined to give consent, those who did not have viral load records in their files, and those who were unable to complete the informed consent or assent process as assessed by the study research assistants (RAs). Trained RAs approached eligible participants on the phone if available or notified the clinic front desk to be contacted if an eligible participant turned up for review. RAs introduced the study to the eligible participant and/or the guardian or parent if they came together at the clinic. The RA then obtained voluntary written informed consent from all eligible participants in the local language within a private area of the hospital. Those who could not write made their thumbprint on the consent form. The RAs obtained permission from a guardian or parent who came with participants below 18 years of age to the health facility. A total of 88 YLWH completed study procedures.

A subset of 15 YLWH was purposively selected from the total enrolled individuals for qualitative interviews based on study participant's family relationship dynamics, viral load outcome, ART enrolment duration, experience, HIV disclosure status, social support characteristics, and variations in the types and quality of social support provided by family members. In addition, participants were invited to come to the clinic or private research space alone. The interviews aimed at gathering in‐depth information from specific participants with characteristics that would help explain their experiences and the role of family support on their ART uptake and utilization.

### 2.4 Data collection

Participants completed a structured interviewer‐led questionnaire with data on known explanatory factors that affect ART uptake and adherence: socio‐demographics, health and depression,^16^ HIV sero‐status disclosure, food insecurity,^11^ alcohol use,^17^ HIV stigma,^18^ and social support.^19^ The questionnaire also contained sections on the last viral load recorded over the previous 6 months, relationship and communication with members at home, income, disclosure status, presence of an HIV‐positive family member, the number of people at home, and family support. Our primary outcome of interest was viral load suppression, defined as a viral load of less than or equal to 500 copies/mL. We expressed the quality of family support as emotional (psychological and informational) and instrumental (physical and economic) social support obtained from family members using a standardized score ranging from 1 to 4,^19^ with 4 indicating high levels of social support.

An in‐depth interview was administered to 15 purposively selected YLWH, which explored family and primary caretaker relationships, ART and disclosure experiences, pill‐taking behavior, food insecurity, and type and variations of family support. The interview guide was developed using the Health Utilization Model (HUM).^20^ All qualitative interviews were conducted within 1 week of participant enrolment by two trained RAs and were digitally recorded with the participant's permission in the native local language (Runyankole) in a comfortable and private location within the Health Center premises or a mutually agreed personal space. Interviews lasted between 45 minutes to 1 hour. The recorded interviews were transcribed from the local language directly to English by a well‐trained RA.

### 2.5 Data analysis

We considered all the 88 YLWH who completed all study procedures. We described demographic and clinical data for the enrolled participants using standard descriptive statistics. We assessed participant correlates of poor viral load suppression of viral load ≤500 copies/mL, computed for each participant. All data analysis was performed using the Statistical Package for Social Sciences (SPSS) version 20. We estimated the *P* values with Chi‐squared tests utilizing a level of statistical significance of ≤.05. Continuous variables were summarized using medians and interquartile ranges. We used univariate logistic regression to assess unadjusted associations between covariates and viral load suppression, and these were expressed using crude odds ratio (OR) and 95% confidence intervals (CIs). We tested the variables for collinearity. Those with a *P* value of less than or equal to .10 in unadjusted analyses were included in a multivariable logistic regression analysis, adding one at a time to control for confounders.

Interviews were transcribed in English and coded manually. Coding was jointly done by ECA and RN. Together with others, disagreements in coding were resolved to ensure consistency. We reviewed the coded data to identify repeated patterns and sorted them to derive categories using the inductive content analytic approach.^21^ We aimed to construct categories describing individual healthcare experiences, relationship dynamics, involvement and perspectives of family to support healthcare decisions, ART uptake and utilization, as well as barriers and challenges that affect their well‐being. Themes were then generated from the categories identified and presented with illustrative quotes from the participants' interviews to explain how these relationships and support—or lack thereof—shape their healthcare decisions and access to and utilization of ART care.

### 2.6 Ethics and approval

This study was approved by the Mbarara University of Science and Technology Research Ethics Committee (MUREC1/7) and the Uganda National Council of Science and Technology (RESCLEAR/01). In addition, the team obtained approvals from the District Health Officer of Rwampara District and the facility in‐charge of Kinoni Health Centre IV before conducting the research.

## 3 RESULTS

### 3.1 Quantitative data

#### 3.1.1 Socio‐demographic characteristics of the study population

This study was initiated in March 2020 and ended in September 2020. A total of 88 study participants were enrolled and interviewed. The median age was 21 (IQR: 16‐24) years, and almost half of this population was aged 23 to 24 years, with 68% having attained at least primary education (Table 1). Half of the interviewed youth (51%) were male. Fifty‐seven percent resided more than 5 km away from the health center where they accessed their routine ART care. Sixty‐eight percent of these youth reported some form of employment. About 39% of the interviewed participants reported hazardous alcohol intake, with a quarter YLWH reporting moderate to high stigma with a median of 3 (IQR: 1‐6.3) on an 8‐point scale. We detected moderate to high depression in 42% of all the enrolled youth. About half of participants (53%) stayed with a parent, sibling, or spouse. More than a third of the participants (34%) had not disclosed their status to any person they stayed with at home. Almost half of the participants (48%) reported having an HIV‐positive family member, with 82% reporting to be staying with more than three people below 18 years of age (Median of 4; IQR: 2‐7). Less than a quarter of the participants (23%) reported having moderate to high family social support (median score 2.3; IQR: 1.6‐3.2). Of those who did not live with a family member, less than half (41%) communicated with any family member at least twice a week. The majority of the youth (74%) sought physical or financial help from family members, whereas most (81%) sought advice from friends instead. Most YLWH (61%) perceived support from family members in the last 2 years as helpful, and less than half (48%) were happy with their current family relationship.

**TABLE 1:** Socio‐demographic, clinical, and family characteristics of study participants

Category	Group	Frequency (%)
Participant characteristics		
Age (y), median (IQR)	21 (16‐22)	
Age	15‐18	25 (28.4)
	19‐22	25 (28.4)
	23‐24	38 (43.2)
Sex	Female	43 (48.9)
Education	≥Primary schooling	60 (68.2)
Distance from clinic	<5 km	36 (40.9)
Employment status	Employed	60 (68.2)
Hazardous alcohol intake	Yes	34 (38.6)
HIV stigma^a^	None	66 (75.0)
	Moderate	13 (14.8)
	High	9 (10.2)
Median HIV stigma score	—	3 (1,6.3)
Depression^b^	No	51 (58.0)
	Moderate	17 (19.3)
	High	20 (22.7)
Family characteristics		
Relationship of the member with whom one lives	Parent/sibling/spouse	47 (53.4)
	Friend/other	41 (46.6)
The primary caretaker has a regular income	Yes	36 (40.9)
Disclosure status to any family member	Yes	58 (65.9)
Known HIV‐positive member in the family	Yes	42 (47.7)
Number of people ≤18 y with whom one stays	≥3	72 (81.8)
The median number of people <18 y staying with IQR		4 (2,7)
Family support^c^	Low	46 (52.3)
	Moderate	23 (26.1)
	High	19 (21.6)
Median social support score	—	2.3 (1.6‐3.2)
Communication with any family member^d^	Daily	8 (19.5)
	At least twice a week	11 (21.9)
	Once a week	7 (17.1)
	>A week	10 (24.4)
	Never	5 (12.2)
Whom you seek/get physical or financial help^e^	Family	65 (73.9)
	Friends/other	33 (37.5)
Whom you talk to for advice^e^	Family	47 (53.4)
	Friends/other	71 (80.7)
	Healthcare provider	63 (71.6)
Perceived family support as helpful	Yes	54 (61.2)
Family relationship satisfaction	Happy	42 (47.7)

^a^ Score for stigma ranges 1‐8, with 8 indicating high levels of HIV stigma.

^b^ Score for depression ranges 0‐10, with 10 showing high levels of depression.

^c^ Social support score ranges 1‐4, with 1 representing low level of support.

^d^ Proportion of those who do not stay with family.

^e^ more than one response.

### 3.2 Patterns of viral load suppression among study participants

All participants were enrolled in the ART clinic and received HIV care at the Kinoni Health Center IV. Up to 78% of YLWH reported viral load suppression. The youth in the age group 19 to 22 years registered the highest viral load suppression (calculated as a percentage per category). Although nonsignificant, females and participants who stayed closer to health facilities adhered to ART more than males (Table 2). Participants who were assessed with no depression recorded higher levels of viral load suppression (94% vs 57%, *P* = .002). Participants who recorded no to low HIV stigma levels also had better viral load suppression (94% vs 32%, *P* = .011). The majority of the participants who stayed with their parent, sibling, or spouse registered a higher viral load of ≤500 copies/mL per category than those who stayed with friends, alone, or other relations such as employer (87% vs 68%, *P* = .031). Better viral load suppression was observed for participants who had disclosed to any family member (86% vs 63%, *P* = .013). Participants reporting moderate to high family support registered higher viral load suppression rates than those with low to no support (87% vs 65%, *P* = .009). Higher rates of viral load of ≤500 copies/mL were also reported among participants who perceived family support as helpful (87% vs 65%, *P* = .001).

**TABLE 2:** Patterns of viral load suppression across different socio‐demographic, clinical, and family characteristics of study participants, N = 88

Category	Group	Frequency of viral load ≤500 copies/mL (%)	Frequency of viral load >500 copies/mL (%)	*P* value
Viral load suppression	≤500 copies/mL	69 (78.4)	19 (21.6)	—
Participant characteristics				
Age	15‐18	19 (76.0)	6 (24.0)	.724
	19‐22	21 (84.0)	4 (16.0)	
	23‐24	29 (76.3)	9 (23.7)	
Sex	Female	37 (86.0)	6 (14.0)	.074
	Male	32 (71.1)	13 (28.9)	
Education	At least primary	46 (76.7)	14 (23.3)	.561
	None	23 (82.1)	5 (17.9)	
Distance from the clinic with 5 km	<5 km	30 (83.3)	6 (16.7)	.059
	≥5 km	39 (75.0)	13 (25.0)	
Employment status	Unemployed	22 (25.0)	6 (21.4)	.980
	Employed	47 (53.4)	13 (21.7)	
Hazardous alcohol intake	Yes	24 (70.6)	10 (29.4)	.157
	No	45 (83.3)	9 (16.7)	
Depression	Yes	21 (56.8)	16 (84.2)	.002
	No	48 (94.1)	3 (5.9)	
Stigma	Moderate‐to‐high	7 (31.8)	15 (68.2)	.011
	No‐to‐Low	62 (93.9)	4 (6.1)	
Family characteristics				
Relationship of people with whom they live	Parent/spouse/sibling	41 (87.2)	6 (12.8)	.001
	Friend/others	28 (68.3)	13 (31.7)	
Disclosure status to a family member	Yes	50 (86.2)	8 (13.8)	.013
	No	19 (63.3)	11 (36.7)	
HIV‐positive family member	Yes	34 (81.0)	8 (19.0)	.580
	No	35 (76.1)	11 (23.9)	
Number of people at home	<3	11 (68.8)	5 (31.2)	.299
	≥3	58 (80.6)	14 (19.4)	
The primary caretaker has a regular income	Yes	30 (83.3)	6 (16.7)	.001
	No	39 (69.2)	13 (25.0)	
Communicates with family at least twice a week^a^	Yes	61 (92.4)	5 (7.6)	.042
	No	8 (36.4)	14 (63.6)	
Family support	Moderate‐to‐high	37 (88.1)	5 (11.9)	.009
	No‐to‐low	32 (69.6)	14 (30.4)	
Perceived family support as helpful^b^	Yes	47 (87.0)	7 (13.0)	.001
	No	22 (64.7)	12 (35.3)	

^a^ Includes those who stay with family.

^b^ Collinear with family relationship satisfaction.

Bivariate analyses identified several factors that were associated with viral suppression (Table 3). Viral load suppression was associated with living with a parent, sibling, or spouse (OR: 3.17; CI: 1.08‐9.34; *P* = .001), having a primary caretaker with a regular income (OR: 1.30; CI: 1.12‐3.50; *P* = .001), and living or communicating with family at least twice a week (OR: 3.93; CI: 1.73‐6.07; *P* = .042). Other significant factors included youth receiving moderate to high family support (OR: 4.60; CI: 1.38‐15.27; *P* = .009) and those who perceived family support in the last 2 years as helpful (OR: 2.15; CI: 1.42‐4.87; *P* = .001). Nondisclosure of the HIV status to family members (OR: 0.28; CI: 0.79‐0.96; *P* = .013), depression (OR: 0.58; CI: 0.21‐0.91; *P* = .002), and moderate to high HIV stigma (OR: 0.26; CI: 0.16‐0.87; *P* = .011), on the other hand, were associated with diminished viral load suppression among participants.

**TABLE 3:** Possible facilitators and barriers to adherence

Category factors	Crude odds ratio (95% CI)	*P* value	Adjusted odds ratio (95%CI)	*P* value
Participant characteristics				
Age (y)				
≥18	Ref. (1.0)			
<18	1.14 (0.36‐3.60)	.819	N/A	N/A
Gender				
Female	Ref. (1.0)		Ref. (1.0)	
Male	0.63 (0.23‐1.76)	.074	0.35 (0.08‐1.56)	.167
Education level				
≥Primary	Ref. (1.0)			
<Primary	0.71 (0.23‐2.23)	.561	N/A	N/A
Distance to nearest health center (km)				
≥5	Ref. (1.0)		Ref. (1.0)	
<5	1.67 (0.57‐4.89)	.059	11.66 (0.38‐98.39)	.124
Employment status				
Unemployed	Ref. (1.0)			
Employed	1.01 (0.34‐3.02)	.980	N/A	N/A
Alcohol use				
Non hazardous	Ref. (1.0)			
Hazardous alcohol use	0.48 (0.17‐1.34)	.157	N/A	N/A
Depression				
None	Ref. (1.0)		Ref. (1.0)	
Moderate to high	0.58 (0.21‐0.91)	.002	0.31 (0.06‐0.52)	.041
HIV stigma				
None	Ref. (1.0)		Ref. (1.0)	
Moderate to high	0.26 (0.16‐0.87)	.011	0.10 (0.02‐0.23)	.007
Family characteristics				
Who one lives with				
Friends/others	Ref. (1.0)		Ref. (1.0)	
Parent, sibling, spouse	3.17 (1.08‐9.34)	.001	6.45 (1.16‐16.13)	.033
Disclosed to any family member				
Yes	Ref. (1.0)		Ref. (1.0)	
No	0.28 (0.96‐0.79)	.013	0.32 (0.06‐1.65)	.174
HIV‐positive family member				
No	Ref. (1.0)			
Yes	1.34 (0.48‐3.73)	.580	N/A	N/A
Under 18 living in a household				
<3	Ref. (1.0)			
≥3	1.89 (0.56,6.30)	.299	N/A	N/A
Income status				
No regular income	Ref. (1.0)		Ref. (1.0)	
Regular income	1.30 (1.12‐3.50)	.001	1.57 (1.09‐4.17)	.014
Lives or communicates with family				
<Twice a week	Ref. (1.0)		Ref. (1.0)	
≥Twice a week	3.93 (1.73‐6.07)	.042	4.12 (1.65‐7.14)	.003
Family support				
None	Ref. (1.0)		Ref. (1.0)	
Moderate to high	4.60 (1.38‐15.27)	.009	12.11 (2.06‐17.09)	.006
Perceived family support				
Not helpful	Ref. (1.0)		Ref. (1.0)	
Helpful	2.15 (1.42‐4.87)	.001	1.98 (1.34‐3.44)	.001

In the multivariate model, living with a parent, sibling, or spouse (AOR: 6.45; CI: 1.16‐16.13; *P* = .033), primary caretaker having a regular income (AOR: 1.57; CI: 1.09‐4.17; *P* = .014), living or communicating with family at least twice a week (AOR: 4.2; CI: 1.65‐7.14; *P* = .003), moderate to high family support (AOR: 12.11; CI: 2.06‐17.09; *P* = .006), and reporting family support received in the last 2 years as helpful (AOR: 1.98; CI: 1.34‐3.44; *P* = .001) were associated with increased odds of viral load suppression. On the other hand, moderate to high HIV stigma (AOR: 0.10; CI: 0.02‐0.23; *P* = .007) and depression (AOR: 0.31; CI: 0.06‐0.52; *P* = .041) were associated with decreased adjusted odds of viral suppression. Disclosure status to family members was no longer significantly affecting the viral load.

### 3.3 Qualitative findings

Our qualitative data showed that youth desired ongoing social and family support to cope with their daily emotional, physical, and economic needs. Most participants depended on family for food, shelter, housework, encouragement, and financial support to refill and take medications daily and timely as needed. Our data also showed the following affected the quality of active family support: (a) dysfunctional family relationships that affected regular communication and contact; (b) economic insecurity and physical separation that affected resource mobilization and food security; (c) fear of disappointment, judgment, and discrimination from family if they disclosed their HIV sero‐status; (d) previous and ongoing family experience with HIV and or ART, which influenced awareness and perceived usefulness of ART. The absence of active family support often fueled a sense of stress, anxiety, abandonment, helplessness, depression, and the desperation to keep their status a secret to remain included in family and community activities, which often left participants feeling alone.

### 3.4 Dysfunctional relationships affecting regular communication and contact

Most participants reported dynamic relationships at the individual, family, and community levels that changed over time, affecting their emotional and physical well‐being. Many participants lived and depended on their relatives, spouses, or friends for food, money, housework, shelter, advice, or emotional support. Others stayed alone because of the death or separation of parents, family conflicts, and their perceived need to be independent and start their own families. Participants often expressed a great desire for continuous physical and emotional support from their close friends, parents, and siblings to constantly communicate challenges, seek advice, encouragement, and cope with disclosure effects. The support rendered also included helping them financially or physically to pick and take their medicines on time. Owing to the inability to get the desired support from immediate family due to mistrust, dysfunctional relationship, and communication, some participants chose to date or get married early to partners perceived to financially and emotionally support them. According to some participants, these complex and dysfunctional relationship arrangements often led to separation, fueling feelings of abandonment, emotional strife, helplessness, self‐neglect, low self‐esteem, anxiety, and depression. Some participants, for example, who stayed with partners who did not know their HIV sero‐status reported experiences of bearing abuse, mistreatment, and loneliness within these relationships to remain and get financial and physical provisions. These challenges often affected pill‐taking behavior, as individuals struggled to pick up their refills on time. One of the participants with a high viral load, who also reported a low family support score, said:

“My family members abandoned me after I lost both my parents (to HIV) to fight for myself. I don't trust them, and I am on my own now … they gossip about me behind my back that I am sick (HIV) and useless and this disturbed me a lot … I got a girlfriend who is much older than me and working (laughing) who cares for me … I get anxious and scared when I miss (picking) my medicine when she's not around to help me and I cannot even call or contact my family.”

Another 18‐year‐old female with poor viral suppression and low family support score said:

“I was very young when I got married to this man after I lost my mother and my father re‐married and abandoned us with our grandmother in the village … Life became difficult. With no food or money for school. We couldn't call anyone for help … I don't trust our stepmother, and she cannot even help. I was forced to marry an older man, but I did not know he was HIV positive … I do not love him. Sometimes he comes home drunk and beats me like a mad man after a small argument. I feel helpless. I feel hopeless … I worry a lot when I fail to pick my medicines in time and cannot tell him to avoid another fight, but I still have to remain with him because he provides for me, my grandmother and my siblings.”

However, a 17‐year‐old female who reported good family support and undetectable viral load said:

“I would love my friends to be more understanding and support me, but I cannot tell them yet … My parents and brothers are good to me and call at all times to check on me. They advise and support me a lot. For example, when I had stopped taking these medicines (ARVs), they were very patient with me and encouraged me alot. They make sure I eat on time, and they escort me to the clinic to get my medicines and sometimes help me pick my medicines like when I am at school.”

### 3.5 Economic insecurity and physical separation affecting resource mobilization

Families helped participants to mobilize basic needs such as food, transport, personal needs, and shelter. Individuals who were entirely dependent on economically secure families reported no worries concerning feeding and upkeep. They also reported experiencing emotional support, as they did not consider themselves a burden to other family members and consistently picked and used their medication as advised by their healthcare providers. However, some participants reported food scarcity, which affected their pill‐taking schedules, and economic strife, which led them to missing medicine refill dates due to limited resources. For example, the youth that reported physical separation from family because of school, work, relocation, divorce, and demise of a parent or guardian struggled to mobilize enough finances to keep them afloat on their own. In such instances, individuals reported alternative ways of mobilizing resources on their own from their social networks or taking up several odd jobs to feed themselves and their dependents. Others failed to muster money in time for transport to the clinic for refills or to secure meals in time and so skipped medication fearing serious side effects. One of the 24‐year‐old female participant who stayed alone and reported a high viral load said:

“My in‐laws have no food, and my husband works at construction sites far away from home and he's always away. So I sometimes sleep hungry, and can't take my medicines because I depend on my husband for food and other financial provisions … I used to get some money for my needs from my brother because my father died and mother does not work anymore, but we relocated and they don't know where I am. I run away to get married to this man and it's a struggle … Sometimes, I have to do small jobs to get some money for good food or otherwise I will miss my medicines because they make me feel so weak and dizzy when I take them without food.”

A 19‐year‐old male who reported good family support with an undetectable viral load said:

“My maternal uncle takes good care of my mother and I … I used to have many challenges when my dad died five years ago, but we stay with him and he's been very supportive, and he helps me go to the clinic whenever I am needed. He pays my tuition as well, so I am not worried at all.”

### 3.6 Fear of disappointment, judgment, and discrimination from family affecting active support

Many participants indicated that they desired their families to support them emotionally and be their first choice source of encouragement and advice whenever they encountered any challenges. Others depended heavily on family members for comfort and guidance every day. However, disclosure to family members about their positive HIV sero‐status was difficult for some participants, especially those who had recently acquired HIV, partly because they felt a sense of guilt and failure on their part to live as expected of their family or community. Such participants primarily reported acquiring HIV as their fault and anticipated feelings of disappointment from their families. These participants also reportedly feared that relatives and or community members would harshly judge them and would be excluded from freely interacting with the family or community if they disclosed their HIV status. With the anticipation of being discriminated against, individuals chose to keep their status a secret. They ended up living away from the prying eyes of their parents, relatives, or friends. This seclusion often led to stress, anxiety, loneliness, and depression, with their family members unaware of what they were going through. These uncertainties contributed to missed medication, especially if they slept out of their primary residences or entertained visitors at home who stayed over past their dosing time. At other times, no one would be there to encourage or remind them to take medication, go for refills, or escort them to the hospital whenever they felt weak or needed emotional support. Some participants, therefore, chose to disclose only to non‐family members or housemates to mitigate some foreseeable challenges. Our data also revealed that participants who had acquired HIV at birth were well counseled over time and found no trouble disclosing to other family members. This confidence was attributed to the feeling that the family was already a part of their journey and was better placed to understand and help them with their health problems and emotionally support them if the need arises. Persons who had been infected from childhood also reported active family support that helped them overcome moments where their peers bullied, underlooked, or mistreated them, especially at school. According to a 20‐year‐old female who reported a moderate family support score but with a high viral load said:

“I stay alone now and find it extremely hard to live by myself as I feel very lonely … I am anxious all the time, and I get scared if my family found out I was HIV‐positive. If I tell them, they will be disappointed and start to treat me differently. They will judge me, and I will no longer be admired by my siblings, mother, or anyone in my village because I messed up … I depend on my family and friends for encouragement, but I cannot disclose to them yet … Sometimes when I have them visiting me, I can't take these medicines because I fear they will see them.”

Another 17‐year‐old male with a suppressed viral load who also reported high family support added:

“I rely on my family for finances, school fees, advice, and encouragement. I used to be bullied at school, children would not want to play with me and maltreated me, and they used to say I would make them sick. I ended up failing to concentrate in class. One day my mother came to school and almost beat up the children that had made fun of me at assembly and explain to them that anyone can get HIV … I was happy and encouraged by her support. She made me feel good and normal again and that I can be with anyone despite my HIV status.”

### 3.7 Family experience with HIV/ART influencing awareness and perceived usefulness of ART

Previous and ongoing family experiences or engagements with HIV‐positive individuals on ART were reported to improve individual's awareness and perceived usefulness of ART, whereas inexperience and information gaps on the other end affected perceived usefulness of ART and uptake. Participants whose close family members had had experience taking ART, taking care of other HIV‐positive family members, or had experiences of relatives who got severely ill and died as a result of defaulting of their ART were scared of meeting a similar outcome. This family attitude, plus a perceived benefit from ART, motivated individuals to continue picking and taking their medications on time as prescribed. Most participants also seemed knowledgeable about their medicines, their use, and their side effects through information obtained from family members enrolled on ART, healthcare providers, or through their community social networks. However, participants who did not have HIV‐related family experiences or adequate knowledge concerning HIV and ART medication did not fully appreciate the usefulness of timely usage of drugs and often seemed detached from the effects of their non‐adherent behavior. Such individuals reported several excuses as to why they could not take their medication on time as prescribed, for example, work or medicine‐related side effects. A 16‐year‐old male who had an undetectable viral load, with moderate family support said:

“I know many people, some in my family, who have died because they did not take their medicines properly. Everyone in my family talks about it a lot and always reminds me about an uncle who also died similarly. It wasn't a good experience for anyone because he was a breadwinner. I always remember to take my medicines because I am scared and know very well that if I don't, I will become very sick and die (sooner) just like my uncle.”

According to a 19‐year‐old male who lives alone, scored moderately on family support, with a very high viral load said:

“It's a new experience for my family and me. Sometimes I forget to take these medicines because these pills are so big and make me feel weak (silence). Sometimes they make me want to vomit, so when I return late, I go to sleep and at times have no time to go to the clinic because I work all night (in a bar) and I am so tired in the morning, so I sleep.”

## 4 DISCUSSION

Our study's main objective was to describe family support patterns and their role on viral load suppression among YLWH aged 15 to 24 years in rural southwestern Uganda. Our findings showed that half of the participants stayed with a family member, 34% had not disclosed their status to any person they stayed with, only 23% reported getting moderate to high family social support, and up to 78% of YLWH reported viral load suppression. Our data also showed that living with a parent, sibling, or spouse (AOR: 6.45; CI: 1.16‐16.13; *P* = .033), the primary caretaker having a regular income (AOR: 1.57; CI: 1.09‐4.17; *P* = .014), individuals who lived or communicated with family at least twice a week (AOR: 4.2; CI: 1.65‐7.14), *P* = .003), reporting good family support (AOR: 12.11; CI: 2.06‐17.09; *P* = .006), and reporting family support received in the last 2 years as helpful (AOR: 1.98; CI: 1.34‐3.44; *P* = .001) were associated with increased adjusted odds of viral load suppression. HIV stigma, on the other hand (AOR: 0.10; CI: 0.02‐0.23; *P* = .007), and depression (AOR: 0.31; CI: 0.06‐0.52; *P* = .041) among YLWH were associated with decreased adjusted odds of reporting viral loads ≤500 copies/mL. Nondisclosure reduced the odds of viral load suppression, although its effect was muted in the multivariate model. Our qualitative data showed that most participants depended on family for food, shelter, housework, encouragement, and financial support to refill and take medications daily and timely as needed. The absence of family support, mainly due to dysfunctional relationships that affected communication and contact, economic insecurity, and physical separation that affected resource mobilization, fueling feelings of strife, abandonment, helplessness, and depression ultimately affected medicine uptake and use. HIV‐related stigma and discrimination at an individual, family, and community level also affected early disclosure and adherence. Previous and ongoing family experiences or engagements with HIV‐positive individuals on ART or HIV care seemed to influence an individual's perceived usefulness of ARV drugs and encourage their use. Inexperience and information gaps, on the other hand, affected the uptake and use of ART.

Previous studies have documented the importance of family dynamics in the mental health of these young adults, indicating that the familial context in which a person with HIV on ART resides is interconnected with their health outcomes.^22^ It has also been shown that providing people with information regarding the myths and the usefulness of ART improves their adherence.^23^ Other studies have shown a high rate of ART adherence among the youth with HIV from cohesive families.^12^ Therefore, the youth mainly rely on their family's social support to enable them to follow the treatment plan offered and cope emotionally and financially with having to take their medication correctly and on time.^24^ Good relationships and social support also improve early linkage to health services, positive living, HIV status disclosure, and self‐acceptance of the HIV status.^25^ Indeed, our data showed that individuals who had relocated from their families for various reasons and experienced challenges communicating with family members about their HIV status, adherence challenges, and emotional strife reported missed doses or visits. In line with previous studies,^26^ our data also showed that the lack of proper food or proper and timely food preparation affected ART use, as individuals tried to avoid the side effects of ART.

Some studies have observed that males, especially the adolescent youth, are less likely to test and or disclose their HIV status to others, including close family members, because of the fear of being discriminated against.^7^, ^27^ These young people also avoided disclosure to those outside their homes because of perceived stigma and discrimination.^28^ On the other end, disclosing one's HIV status and receiving acceptance and social support from close relations were associated with improved long‐term quality of life among the youth.^15^ Our data showed that individuals who had acquired HIV at birth experienced automatic exposure to family members over time and obtained the necessary family support to continue disclosing to significant others. Participants who disclosed earlier had the needed continuous HIV care over time, especially among those whose family relationships were good. Incoherent family relationship, on the other hand, facilitated emotional strife and fear of victim‐blaming following disclosure, and this affected pill‐taking behavior, regular refills, and adherence among participants. As previously reported,^29^ the lack of physical and emotional support from close families of the HIV‐infected youth significantly affected their mental and physical well‐being.

### 4.1 Strengths and limitations

Our study had several strengths. First, ours was one of the few studies, to the best of our knowledge, that used a mixed‐method approach to document the patterns of family support and its primary role in viral load suppression among YLWH. The location being in rural southwestern Uganda, a cultural setting where family relationships and dynamics are key, gives a better explanation of their effects on life choices and health outcomes of YLWH. Therefore, our study contributes to a greater understanding of the characteristics and complexities of families and family relationships that may influence medication‐specific adherence. Second, we collected our data from youth accessing HIV care at Kinoni Health center IV, a publically funded and operated health center in a rural setting with an active HIV clinic, subject to standard limitations of public sector healthcare facilities in the region and diverse healthcare users. Our results documented dynamic family relationships that affected social support, disclosure, and adherence to ART. Therefore, our data can inform interventions aimed at improving uptake and adherence to HIV services by incorporating and engaging communities on the role of family support on disclosure, compliance to HIV care, and or ART adherence. Our study also had limitations. We used a small sample of 88, which may have overestimated the odds ratios in logistic regression or limited the ability to explore the association between other participant or family characteristics and adherence fully. A more extensive study in diverse populations may be needed to adequately assess the role of family support on adherence among youth in such a setting.

## 5 CONCLUSION

Half of YLWH stayed with a family member, 34% had not disclosed their status to any person they stayed with, and only 23% reported getting moderate to high social support from family. Up to 78% of YLWH had a viral load of ≤500 copies/mL. Our data showed that living with a family member, living with a primary caretaker with regular income, living or communicating regularly with family at least twice a week, and individuals who reported good family support significantly increased the odds of viral load suppression. Experiencing depression and HIV‐related stigma decreased the odds of viral load suppression. Qualitative data showed that all YLWH desired regular and ongoing emotional, physical, and financial support from their immediate family to access and take medications daily and timely as needed. However, many of them cannot get adequate active support from their immediate families because of dysfunctional relationships that affected regular communication and contact, economic insecurity, and physical separation that affected resource mobilization, fear of disappointment, judgement, and discrimination from family and friends if they disclosed earlier. Families who previously experienced HIV and ART were more aware of the usefulness of ART and seemed to provide the support individuals needed for ongoing medication uptake and use. The absence of good family support facilitated feelings of abandonment, economic or emotional stress, anxiety, helplessness, desperation, and low perceived usefulness for ARV drugs.

A contextual understanding of community needs and factors that provide an enabling environment to suppress viral load among YLWH is needed to maximize their mental well‐being and ART clinical treatment outcomes. In addition, future studies should explore group family HIV/ART counseling and a community awareness approach to encourage acceptance, resource mobilization, and disclosure for groups who greatly rely and thrive on active family support.

## CONFLICT OF INTEREST

All authors declare no conflict of interest.

## AUTHOR CONTRIBUTION

Conceptualization: Rachel Mukisa Nakandi, Patricia Kiconco, Angella Musiimenta, John Jonnes Bwengye, Richard Kyomugisa, Esther Cathyln Atukunda

Formal Analysis: Rachel Mukisa Nakandi, Patricia Kiconco, John Jonnes Bwengye, Richard Kyomugisa, Esther Cathyln Atukunda

Funding Acquisition: Rachel Mukisa Nakandi, Patricia Kiconco, John Jonnes Bwengye, Richard Kyomugisa, Celestino Obua, Esther Cathyln Atukunda

Investigation: Rachel Mukisa Nakandi, Patricia Kiconco, John Jonnes Bwengye, Richard Kyomugisa

Methodology: Rachel Mukisa Nakandi, Patricia Kiconco, Esther Cathyln Atukunda, Celestino Obua

Writing – review and editing: Rachel Mukisa Nakandi, Patricia Kiconco, Angella Musiimenta, John Jonnes Bwengye, Richard Kyomugisa, Esther Cathyln Atukunda, Celestino Obua

Writing – original draft: Rachel Mukisa Nakandi, Esther Cathyln Atukunda

Supervision: Esther Cathyln Atukunda, Celestino Obua

Validation: Rachel Mukisa Nakandi, Patricia Kiconco, Angella Musiimenta, Esther Cathyln Atukunda, Celestino Obua

All the authors read and approved the final version of the manuscript.

## TRANSPARENCY STATEMENT

I, Dr. Esther Cathyln Atukunda affirms that there has not been any important aspects of the data that have been left out intentionally. I also affirm that the data herein included in this manuscript is accurate and transparent, and that I had full access to all of the data in the study. I take complete responsibility for the integrity of the data and the accuracy of the data analysis.

## Data Availability

All data has been included in this manuscript. Any additional data will be available on request.
